# Supplementary material for: Splice-Junction-Based Mapping of Alternative Isoforms in the Human Proteome
Source: Cell Rep. Author manuscript; Available in PMC 2020 Jan 15. (PMC6961840; doi:10.1016/j.celrep.2019.11.026)
Supplement: 3 [file NIHMS1546469-supplement-3.zip › DF2/PXD009737/TestisGluC-32-Q9NYF8-TAKTGKFLKSPPLHKNLDARE.pdf]

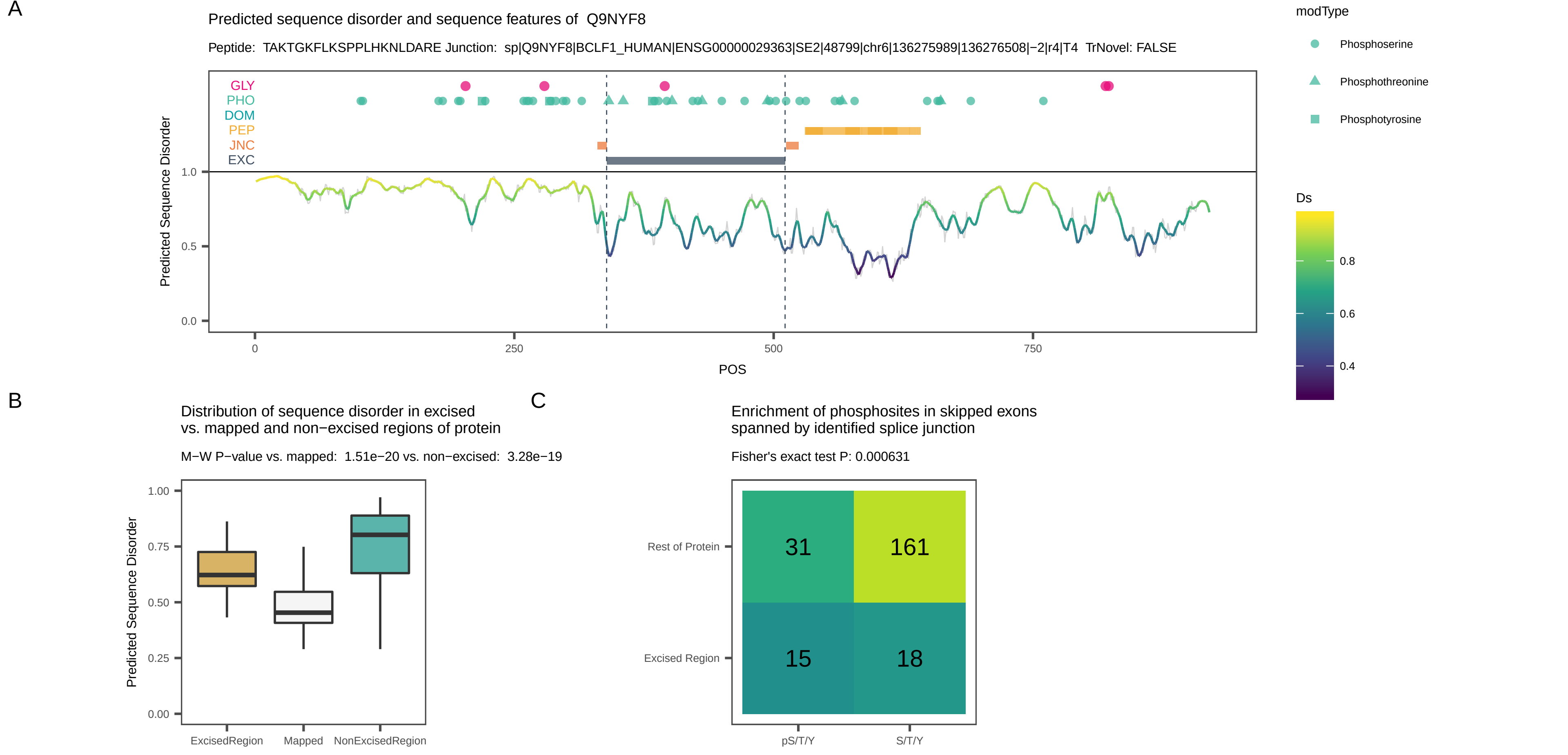
A
Predicted sequence disorder and sequence features of Q9NYF8
Peptide: TAKTGKFLKSPPLHKNLDARE Junction: sp|Q9NYF8|BCLF1_HUMAN|ENSG00000029363|SE2|48799|chr6|136275989|136276508|−2|r4|T4 TrNovel: FALSE
GLY
PHO
DOM
PEP
JNC
EXC
Predicted Sequence Disorder
1.0
0.5
0.0
0
250
500
750
POS
modType
Phosphoserine
Phosphothreonine
Phosphotyrosine
Ds
0.8
0.6
0.4
B
Distribution of sequence disorder in excised vs. mapped and non−excised regions of protein
M−W P−value vs. mapped: 1.51e−20 vs. non−excised: 3.28e−19
Predicted Sequence Disorder
1.00
0.75
0.50
0.25
0.00
ExcisedRegion
Mapped
NonExcisedRegion
C
Enrichment of phosphosites in skipped exons spanned by identified splice junction
Fisher's exact test P: 0.000631
Rest of Protein
Excised Region
31
161
15
18
pS/T/Y
S/T/Y
